# Functional connectivity changes between parietal and prefrontal cortices in primary insomnia patients: evidence from resting-state fMRI

**DOI:** 10.1186/2047-783X-19-32

**Published:** 2014-06-10

**Authors:** Yongli Li, Enfeng Wang, Hongju Zhang, Shewei Dou, Liya Liu, Li Tong, Yu Lei, Meiyun Wang, Junling Xu, Dapeng Shi, Qingyong Zhang

**Affiliations:** 1 Department of Radiology, Henan Provincial People’s Hospital, People’s Hospital of Zhengzhou University, Zhengzhou, Henan 450003, China; 2 China National Digital Switching System Engineering and Technological Research Center, Zhengzhou, Henan, China; 3 Second affiliated hospital of Zhengzhou University, Zhengzhou, Henan, China

**Keywords:** Functional MRI, Primary insomnia, Working memory, Superior parietal lobule

## Abstract

**Background:**

Primary insomnia can severely impair daytime function by disrupting attention and working memory and imposes a danger to self and others by increasing the risk of accidents. We speculated that the neurobiological changes impeding working memory in primary insomnia patients would be revealed by resting-state functional MRI (R-fMRI), which estimates the strength of cortical pathways by measuring local and regional correlations in blood oxygen level dependent (BOLD) signs independent of specific task demands.

**Methods:**

We compared the R-fMRI activity patterns of 15 healthy controls to 15 primary insomnia patients (all 30 participants were right-handed) using a 3.0 T MRI scanner. The SPM8 and REST1.7 software packages were used for preprocessing and analysis. Activity was expressed relative to the superior parietal lobe (SPL, the seed region) to reveal differences in functional connectivity to other cortical regions implicated in spatial working memory.

**Result:**

In healthy controls, bilateral SPL activity was associated with activity in the posterior cingulate gyrus, precuneus, ventromedial prefrontal cortex, and superior frontal gyrus, indicating functional connectivity between these regions. Strong functional connectivity between the SPL and bilateral pre-motor cortex, bilateral supplementary motor cortex, and left dorsolateral prefrontal cortex was observed in both the control group and the primary insomnia group. However, the strength of several other functional connectivity pathways to the SPL exhibited significant group differences. Compared to healthy controls, connectivity in the primary insomnia group was stronger between the bilateral SPL and the right ventral anterior cingulate cortex, left ventral posterior cingulate cortex, right splenium of the corpus callosum, right pars triangularis (right inferior frontal gyrus/Broca’s area), and right insular lobe, while connectivity was weaker between the SPL and right superior frontal gyrus (dorsolateral prefrontal cortex).

**Conclusion:**

Primary insomnia appears to alter the functional connectivity between the parietal and frontal lobes, cortical structures critical for spatial and verbal working memory.

## Background

Chronic insomnia is the most common sleep disorder, afflicting 10 to 20% of the adult population worldwide [1,2]. It is defined by the *Diagnostic and Statistical Manual of Mental Disorders, version 4* (DSM-IV) as both an independent psychiatric syndrome (primary insomnia) and a common comorbidity (secondary insomnia) associated with a variety of physical and psychiatric disorders [3]. According to a global insomnia survey by the Chinese Medical Association (CMA), 42.5% of people in China suffer from occasional insomnia, and rates are increasing due to industrialization, urbanization, and work pressures. Difficulty falling asleep and staying asleep reduces quality of life [4]. The most salient effect of insomnia is increased daytime sleepiness, resulting in impaired concentration, cognition, and memory [5]. Moreover, insomnia patients reported more missed work days and greater utilization of the healthcare system, even when factoring in the higher prevalence of depression and chronic medical conditions [6]. While both pharmacological and behavioral therapies have demonstrated efficacy for the treatment of insomnia, drugs for insomnia carry potential risks of overdose and dependency, while cognitive behavior therapies are expensive, time consuming, and not widely accessible by many sufferers [7]. Development of safer or more practical alternatives requires further insight into the neurobiological changes associated with chronic sleep deprivation.

Sleep is critical for brain development and function [8-10]. Working memory (WM), a core component of executive function, is markedly disrupted by sleep deprivation [11-13]. Neural circuits associated with emotional regulation and expression are markedly altered in insomnia patients as revealed by functional magnetic resonance imaging (fMRI) [14]. Resting- state functional magnetic resonance imaging (R-fMRI) revealed reduced amygdala connectivity to the insula, striatum, and thalamus, as well as enhanced functional connectivity with the pre-motor and sensorimotor cortex, suggesting potential neurological mechanisms for dysregulated emotional control and affective disorders associated with insomnia [15,16]. Sleep deprivation also triggers compensatory mechanisms to overcome fatigue that are manifested by changes in neural connectivity patterns on fMRI [17].

The prevalence of secondary insomnia may be several-fold greater than primary insomnia [18] and symptoms more severe [19]. However, associated neuropsychiatric comorbidities are manifested by a variety of changes in brain physiology and functional organization [20,21], so only studies of primary insomnia can reveal changes in functional connectivity associated specifically with sleep deprivation. Resting-state fMRI (R-fMRI) measures changes in neural connectivity patterns unbiased by the demands of specific cognitive tasks [22,23]. Thus, R-fMRI is optimal for foundational studies on the anatomic and functional alterations associated with insomnia. Moreover, functional connectivity patterns defined by R-fMRI are well correlated with task-dependent activity patterns [24]. As WM is essential for daily function and markedly degraded by sleep deprivation, we examined changes in functional cortical circuits implicated in WM using R-fMRI. The parietal lobe is an important structure for spatial working memory (SWM) as indicated by BOLD signals during SWM tasks in healthy controls [25], and so was chosen as the seed regions for this study.

## Method

The study procedures were approved by the Zhengzhou University ethics committee and all subjects provided written informed consent. We compared the R-fMRI of 15 healthy right-handed undergraduate students or hospital faculty staff from Zhengzhou University Medical School and Hospital (mean age ± SD: 39.8 ± 11.2 years; age range 21 to 65 years, 8 females and 7 males) to age- and gender ratio-matched right-handed subjects meeting DSM-IV inclusion criteria for primary insomnia (41.3 ± 8.9 years; age range 23 to 54 years, 8 females and 7 males) [3]. Both primary insomnia patients and healthy controls were evaluated using the Pittsburg Sleep Quality Index (PSQI) [26]. All healthy controls reported their sleep as restorative and satisfactory, and all had regular sleep habits with PSQI total scores < 5. Both cohorts were also screened to ensure that they had no history of chronic medical or psychiatric illnesses and had never been medicated for insomnia or suffered other sleep disorders. No participant habitually consumed more than 250 mg/day caffeine. All participants were instructed to rest for 30 minutes prior to the experiment and not to consume caffeine, alcohol, or any other psychoactive substances for 24 hours before the R-fMRI study.

### Data Acquisition

Functional imaging was conducted on a Siemens 3.0 T TrioTim whole-body scanner (Siemens AG, Erlangen, Germany) using a 12-channel array coil in the Henan Provincial People’s Hospital.

High-resolution T1-weighted anatomic images were obtained using a 3D-MPRAGE sequence (TR = 1,950 ms, TE = 2.30 ms, Ti = 900 ms, scan time = 4.24 minutes, matrix = 248 × 256, slice thickness = 1 mm, no distance, FOV = 244 × 252) and fMRI images using a BOLD-fMRI sequence (TR = 3,000 ms, TE = 2.50 ms, matrix = 320 × 320, slice thickness = 5 mm, slice interval = 0.5 mm, total layer = 35, FOV = 210 × 210, scan time = 7 minutes). The entire brain was scanned in 140 volumes. Subjects were instructed to keep their eyes closed, relax, and remain calm during the resting-state scan.

### Functional MRI data analysis processing

#### Data pre-processing

Pre-processing was performed using SPM8 (SPM8, http://www.fil.ion.ucl.ac.uk/spm) and REST (Resting-state data analysis toolkit, http://www.restfmri.net/forum/REST) programs. All functional runs were expressed relative to the first values in each run. We set a movement threshold of 1.5 mm and 1.5° for the 3 linear and 3 axial coordinates to eliminate subjects with excessive head movement. However, none of the subjects had head movements that exceeded threshold. All functional runs were normalized to Montreal Neurological Institute (MNI) space with voxel re-sampling to 3 × 3 × 3 mm^3^. After spatial normalization, we used REST to extract the linear changes over time within the 0.01 to 0.08 Hz bandwidth. The resulting time series were then spatially smoothed with a 4-mm full-width at half maximum (FWHM) Gaussian kernel.

### Functional connectivity analysis

The parietal lobe plays a dominant role in spatial information processing [27] and so was chosen as the seed region to determine changes in functional connectivity patterns by R-fMRI. The exact region of interest (ROI) within the bilateral superior parietal lobe was chosen using WFU_PickAtlas (http://www.ansir.wfubmc.edu). Voxels within the seed region were averaged to generate reference time series. For each subject, a correlation map was then produced by computing the Pearson’s correlation coefficients between the reference time series and the time series of all other brain voxels. The Fisher transformation was used to compute the z-values from r-values in order to improve the normality. Individual z-values for each voxel outside the SPL were compared to that of the SPL seed region ROI by one-sample *t*-test to reveal brain regions showing significant connectivity to the bilateral SPL. Clusters larger than 405 mm^3^ with *P* < 0.005 were accepted and used to construct functional connectivity images.

### Statistical analysis

The z-values were also entered into a random effect two sample *t*-test to identify regions showing significant differences in connectivity relative to the bilateral superior parietal lobes between primary insomnia patients and healthy controls. We set *P* < 0.05 as a significant difference threshold.

## Results

**Figure 1:**
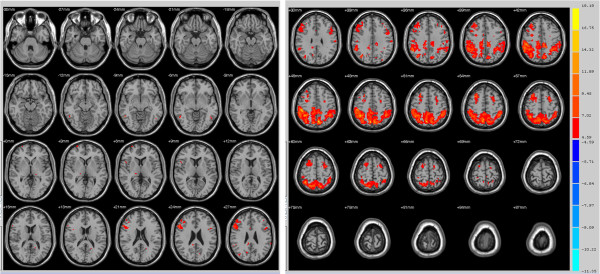
**Maps of resting-state connectivity to the bilateral superior parietal lobe (SPL) in healthy controls.** Colored areas are those regions with a time series correlation relative to the SPL of *P* < 0.005 and minimum cluster size of 405 mm^3^. The right fusiform gyrus, right anterior prefrontal cortex, right somatosensory association cortex, bilateral pre-motor cortex, bilateral supplementary motor cortex, and left dorsolateral prefrontal cortex exhibited strong functional connectivity with the bilateral SPL.

**Figure 2:**
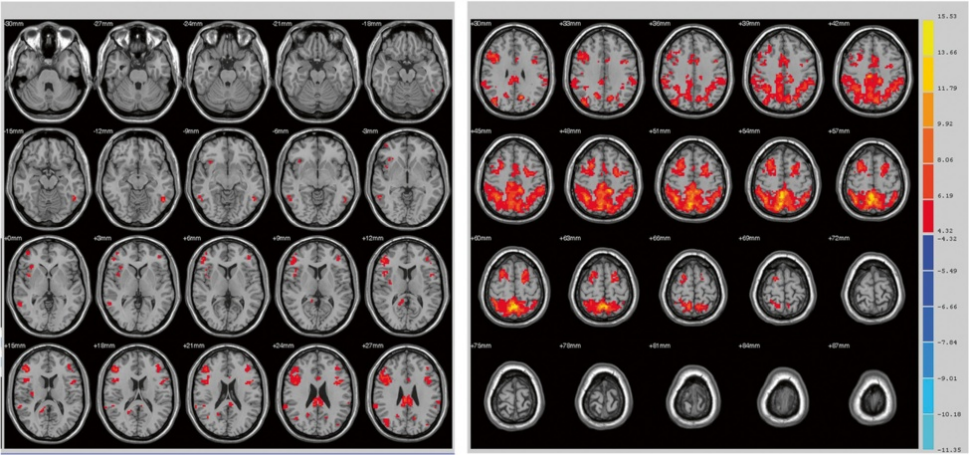
**Maps of resting-state connectivity to the bilateral superior parietal lobe (SPL) in primary insomnia patients.** Colored areas are those regions with a time series correlation relative to the SPL of *P* < 0.005 and a minimum cluster size of 405 mm^3^. The right superior temporal gyrus, right pars triangularis (inferior frontal gyrus/Broca’s area), right dorsolateral prefrontal cortex, right insular cortex, bilateral pre-motor cortex, bilateral supplementary motor cortex, left fusiform gyrus, and left dorsolateral prefrontal cortex exhibited strong functional connectivity with the bilateral SPL.

**Figure 3:**
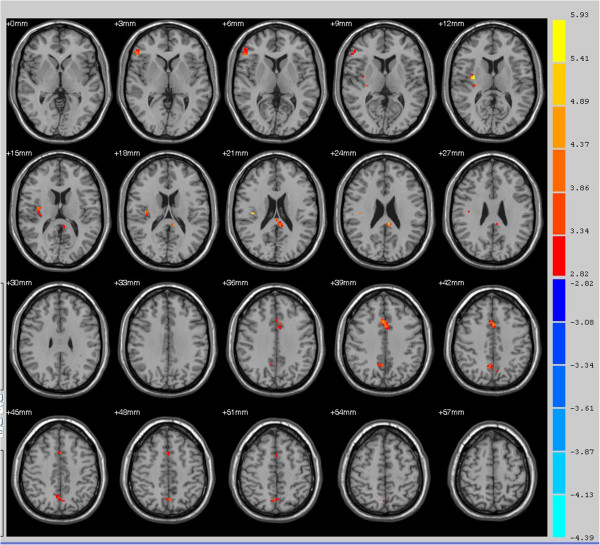
**Areas with higher functional connectivity to the bilateral superior parietal lobe in primary insomnia patients compared to healthy controls.** Areas of greater functional connectivity in patients include the right pars triangularis (inferior frontal gyrus/Broca’s area, thin blue arrow), right insular cortex (thick blue arrow), right anterior cingulate cortex (thin red arrow), and left ventral posterior cingulate cortex (thick red arrow) (all *P* < 0.01).

**Figure 4:**
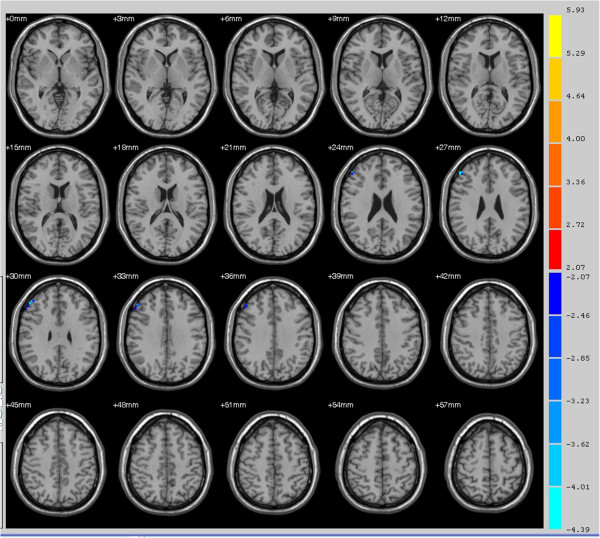
**Areas with lower resting-state connectivity to the bilateral superior parietal lobe in primary insomnia patients compared to healthy controls, including the right superior frontal gyrus (right dorsolateral prefrontal cortex, red arrow) (*P*** **< 0.05).**

We examined differences in connectivity to the bilateral superior parietal lobe (SPL) between a healthy control group and a primary insomnia patient group using resting-state fMRI (R-fMRI). In the healthy control group, there were strong correlations between SPL and posterior cingulate and the nearby precuneus, ventromedial prefrontal gyrus, and superior frontal gyrus (Figure 1). Resting-state activities of bilateral pre-motor cortex, supplementary motor cortex, and left dorsolateral prefrontal cortex were strongly correlated with resting- state SPL activity in both the primary insomnia patient group and the control group (Figures 1, 2). Several regions, however, exhibited significant group differences in functional connectivity to the SPL as revealed by two sample *t*-tests. Compared to the healthy control group, the primary insomnia patient group showed significantly increased functional connectivity between the SPL and bilateral anterior and posterior cingulate, splenium of the corpus callosum, right middle frontal gyrus, and right claustrum (Figure 3 and Table 1). In contrast, the functional connectivity between the SPL and superior frontal gyrus was lower in primary insomnia patients (Figure 4 and Table 1).

**Table 1:** Functional connectivity differences between the healthy and primary insomnia groups

**Cortical regions**	**BA**	**Clusters**	** MNI coordinate**			***t*-values**	***P-*value**
			X	Y	Z		
Right pars triangularis, part of Broca’s area	45	20	54	32	4	3.6824	0.01
Right insular cortex	13	26	33	-7	13	4.7183	0.01
Left ventral posterior cingulate cortex	23	18	-6	-37	25	4.0944	0.01
Right ventral anterior cingulate cortex	24	39	3	17	40	4.0641	0.01
Right dorsal posterior cingulate cortex	31	31	6	-58	43	3.5088	0.01
Right dorsolateral prefrontal cortex	46	21	51	38	28	-4.3949	0.05

BA, Brodmann area; MNI: Montreal Neurological Institute.

## Discussion and conclusions

Current MRI research on primary insomnia has focused on emotional circuit dysfunction [14] and changes in pathways mediating hyperarousal [28]. Huang *et al*. [14] found reduced connectivity of the amygdala to the insula, striatum, and thalamus by R-fMRI, but increase amygdalar connectivity to the pre-motor and sensorimotor cortices in primary insomnia patients. These changes in emotional circuits may contribute to the ultimate development of comorbidities such as depression and anxiety disorder, while the increased functional connectivity of the amygdala to the pre-motor and sensorimotor cortices may be compensatory mechanisms to maintain psychomotor performance under increased daytime fatigue [14]. It has long been speculated that insomnia results from psychological hyperarousal by stressors and, in fact, insomnia patients exhibited enhanced functional connectivity among various sensory cortices and supplementary motor cortex (SMC) as revealed by R-fMRI, and sleep initiation difficulties were associated with the strength of sensory-SMC connectivity [28]. Despite the fact that insomnia is known to markedly impair WM and other aspects of executive function, there have been no studies on possible connectivity changes associated with SWM dysfunction in primary insomnia patients. We demonstrate clearly reduced functional connectivity between the SPL and superior frontal gyrus, which may underlie spatial and verbal WM deficits resulting from insomnia.

SWM tasks activate the hippocampus, the cingulate gyrus, prefrontal dorsolateral cortex, occipitotemporal junction cortex, and parietal cortices [29-34], and SWM depends critically on the interaction between frontal and parietal regions [35]. Indeed, when the superior parietal lobes are damaged, sense of position and directional motion are lost, while unilateral right parietal lobe damage is associated with contralateral neglect [27]. The mammalian parietal medial surface and dorsolateral superior parietal region project to and receive fibers from the prefrontal cortex. The parietal region Brodmann area (BA) 7, which is involved in integration of visual and proprioceptive information, and BA 46 in the prefrontal cortex have particularly strong bilateral connections. The dorsal prefrontal lobe receives association fibers from the parietal lobe encoding dynamic visual information on position and space between objects. In the prefrontal lobe, these inputs are integrated to form spatial working memories that aid in various cognitive processes [36]. In addition to SWM, these two regions are also critical for verbal WM [37].

In our study, we measured strong connections between the SPL and the posterior cingulate gyrus (PCC), precuneus, ventromedial prefrontal cortex, and superior frontal gyrus in healthy controls. These areas were also strongly connected in primary insomnia patients with the exception of the superior frontal gyrus. In contrast, the functional connectivity between the anterior cingulate cortex (ACC) and SPL was stronger in primary insomnia patients. It is agreed that the prefrontal lobe subserves WM and that the superior prefrontal cortex stores information on spatial position [32-34]. Primary insomnia patients had lower functional connectivity between the superior frontal gyrus and SPL, consistent with the SWM deficits observed in these patients. Further research using complementary R-fMRI and fMRI during SWM tasks could reveal additional insights into the neurobiology of WM deficits due to insomnia.

In conclusion, our study strongly suggests that primary insomnia induces memory retrieval deficits in human subjects by disrupting the functional connectivity between the superior frontal gyrus and superior parietal lobe.

## Abbreviations

BA: Brodmann area; BOLD: blood oxygen level dependent; CMA: Chinese Medical Association; fMRI: functional magnetic resonance imaging; FWHM: full-width at half maximum; MNI: Montreal Neurological Institute; PSQI: Pittsburg Sleep Quality Index; R-fMRI: resting- state functional MRI; SPW: superior parietal lobe; SWM: spatial working memory; WM: Working memory.

## Competing interests

The authors declare that they have no competing interests.

## Authors’ contributions

DS, QZ and YL made substantial contributions to study conception and design as well as data acquisition and analysis, drafting the manuscript or revising it critically for important intellectual content, and gave final approval for publication. EW, SD, and LL were involved in data acquisition, analysis, and interpretation. HZ helped screen primary insomnia patients and healthy controls. LT, YL, M W, and JX were involved in analysis and interpretation of data. All authors read and approved the final manuscript.
